# Mechanisms of fibrous cap formation in atherosclerosis

**DOI:** 10.3389/fcvm.2023.1254114

**Published:** 2023-08-21

**Authors:** Laura Alonso-Herranz, Julián Albarrán-Juárez, Jacob Fog Bentzon

**Affiliations:** ^1^ Atherosclerosis Research Unit, Department of Clinical Medicine, Aarhus University, Aarhus, Denmark; ^2^ Steno Diabetes Center Aarhus and Department of Cardiology, Aarhus University Hospital, Aarhus, Denmark; ^3^ Centro Nacional de Investigaciones Cardiovasculares Carlos III, Madrid, Spain

**Keywords:** fibrous cap, atherosclerosis, plaque rupture, smooth muscle cells, clonal expansion, neomedia, arterial media development

## Abstract

The fibrous cap is formed by smooth muscle cells that accumulate beneath the plaque endothelium. Cap rupture is the main cause of coronary thrombosis, leading to infarction and sudden cardiac death. Therefore, the qualities of the cap are primary determinants of the clinical outcome of coronary and carotid atherosclerosis. In this mini-review, we discuss current knowledge about the formation of the fibrous cap, including cell recruitment, clonal expansion, and central molecular signaling pathways. We also examine the differences between mouse and human fibrous caps and explore the impact of anti-atherosclerotic therapies on the state of the fibrous cap. We propose that the cap should be understood as a neo-media to substitute for the original media that becomes separated from the surface endothelium during atherogenesis and that embryonic pathways involved in the development of the arteria media contribute to cap formation.

## 1. Introduction

**Figure 1:**
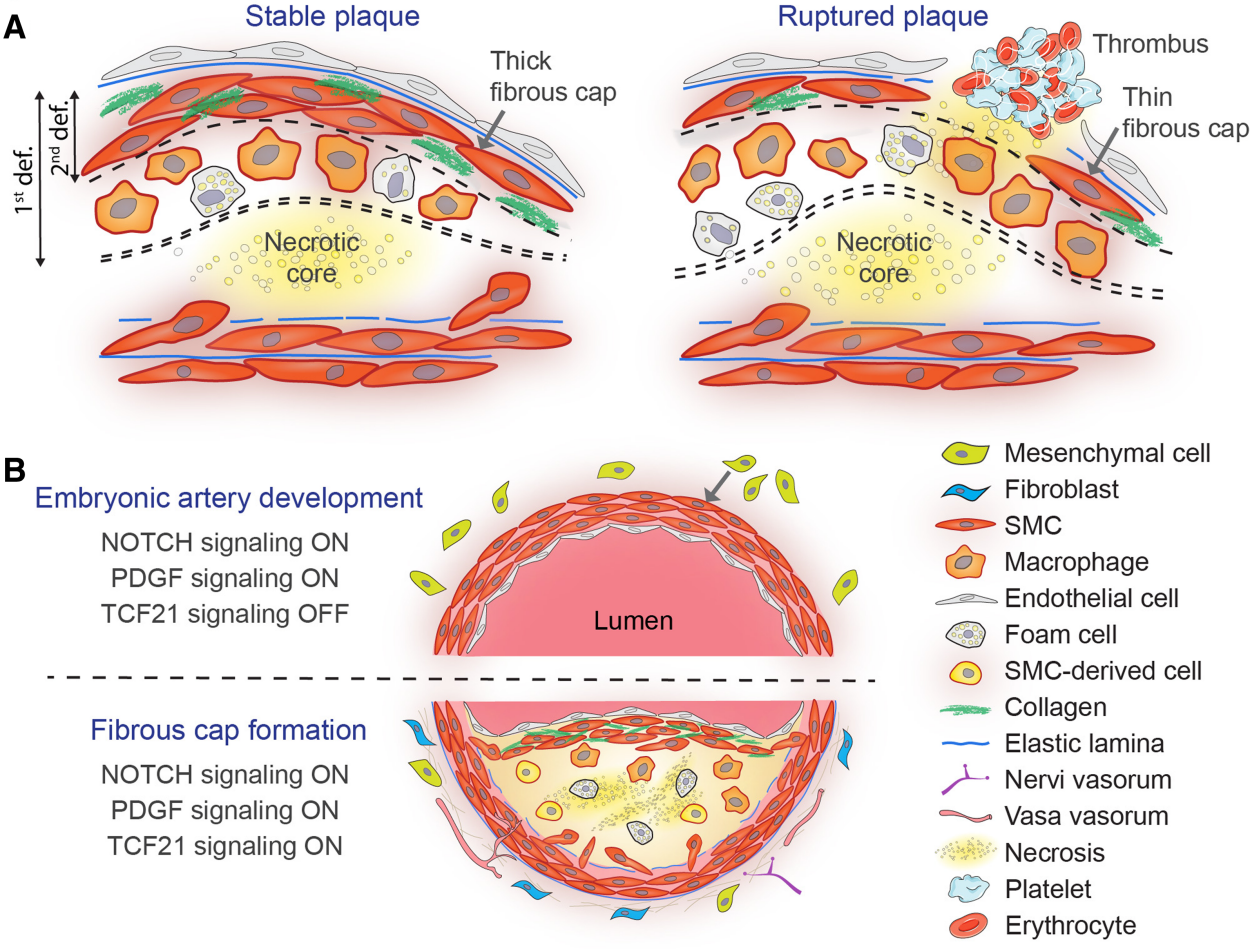
Stable vs. ruptured atherosclerotic plaque (**A**) and similarities between embryonic arterial media development and fibrous cap formation in atherosclerosis (**B**). (**A**) A simplified scheme depicting the media and neointima of a stable murine lesion with a thick collagen-rich fibrous cap overlying a necrotic core (left). The fibrous cap is highlighted by double or single dashed lines according to the first (i.e., space between the endothelium and the necrotic core) or the second definition (i.e., subendothelial SMCs), respectively. In contrast, lesions with thinner fibrous caps and large necrotic cores are at risk for rupturing and precipitating thrombosis (right). (**B**) NOTCH, PDGF, and TCF21 signaling control SMC recruitment and differentiation during mouse embryonic artery development (top) and fibrous cap formation in atherosclerosis (bottom). Mesenchymal precursor differentiation (either from the neural crest, mesoderm, or the epicardium) towards the SMC fate and away from other lineages (i.e., osteochondrocytes and fibroblasts) requires active PDGF and NOTCH signaling (ON) and silencing of TCF21 (OFF). Fibrous cap SMCs are primarily originated by migration and clonal expansion of medial SMCs and blockade of NOTCH, PDGF, or TCF21 signaling is sufficient to impair cap formation.

Each year, millions of deaths occur due to *plaque rupture* of atherosclerotic lesions in arteries supplying the heart or brain (1). Rupture occurs when the *fibrous cap*, which overlies a necrotic core, becomes too weak to withstand the forces of pulsatile blood flow, resulting in its breakage. The rupture may be large resulting in the expulsion of necrotic core material into the bloodstream or be limited to a small tear in the surface of plaques (sometimes called fissure) (2). In both cases, the exposure of core material and collagens may activate thrombocytes and the coagulation system and lead to occluding thrombosis (Figure 1A) (3).

Structurally, the fibrous cap in advanced plaque consists of layers of smooth muscle cells (SMCs) embedded in a collagen and elastin-rich matrix (Figure 1A). The cap SMCs express contractile proteins, such as ACTA2 (actin alpha 2, smooth muscle) and MYH11 (myosin heavy chain 11), but unlike SMCs of the healthy arterial media, the myofilaments are restricted to the cytoplasmic periphery, while the interior is occupied by an abundant endoplasmic reticulum and Golgi bodies, consistent with active extracellular matrix production (4).

In human pathology, the cap is often defined as the tissue that separates a necrotic core from the lumen (4, 5). This definition is meaningful considering the critical importance of this tissue for plaque stability. However, the accumulation of subendothelial SMCs is also found in regions of advanced plaque where no necrosis is present, and in experimental research in mouse models, the fibrous cap is often simply defined as the layer of ACTA2+ SMCs in the subendothelial space. It is helpful to bear in mind that these different definitions exist and are being used interchangeably in the literature on fibrous cap formation.

According to the first definition of the cap, which refers to the tissue separating the necrotic core from the lumen, the cap of human plaques initially consists of the original intimal tissue. As lesions developed into fibroatheromas, this tissue is gradually replaced by a denser SMC-rich layer with more collagen (6). The thickness of the cap tissue is the most important factor determining the risk of rupture, and coronary plaques rarely rupture if it is thicker than 65 µm (7). In mouse models of atherosclerosis, the tissue separating the core from the lumen is often dominated by big macrophage foam cells (Figure 1A), and therefore, its thickness does not provide a clear measure of cap strength.

By the second definition of the cap, which defines it as the layer of subendothelial SMCs (Figure 1A), cap formation serves to restore the supportive layer of SMCs beneath the surface endothelium. This endothelial-SMC connection is present in normal arteries but is temporarily lost or challenged during plaque formation. Notably, the two definitions overlap in human fibroatheroma, the typical precursor of acute coronary events, as the tissue separating necrotic cores consists mainly of subendothelial SMCs and the matrix they produce. This brief review examines cap formation in the sense of the second definition.

The stability of atherosclerotic plaques relies on a delicate balance between the formation and degradation of the fibrous cap. Several previous reviews have focused on cap degradation resulting from SMC senescence and death (8), as well as the breakdown of collagen and other extracellular matrix components (9–11). Here, we examine the fibrous cap from the opposite perspective. We aim to provide an overview of the main mechanisms by which SMCs in experimental models are recruited to the subendothelial space and form the cap. Furthermore, we explore the overlap in underlying molecular mechanisms with embryonic artery development. Finally, we review the effects of therapies and interventions aimed at reducing atherosclerosis on the condition of the fibrous cap.

## 2. Mechanisms of cap formation

### 2.1. Clonal expansion of medial SMCs

SMCs have been classically identified by the expression of contractile proteins defined as SMC lineage markers (e.g., MYH11, ACTA2). However, lineage tracing studies in the past decade have demonstrated that atherosclerotic plaques are dominated by cells with an SMC origin, which have lost the expression of canonical SMC markers (12). These cells are commonly referred as SMC-derived cells.

Multicolored SMC lineage-tracing reporters, such as Rosa26-Confetti or Rosa26-Rainbow mice combined with *Myh11*-Cre^ERT2^ or *Acta2*-Cre^ERT2^ mice, have been used to assess the clonal structure of SMCs investing into plaques (13–16). In these models, labeled cells were found to be organized in large monochromatic patches indicating that SMC-derived plaque cells originate from a limited number of founder SMCs in the arterial media that clonally expand to populate the lesion (13, 14).

Each of these founder SMCs possesses the plasticity to produce plaque cells with different phenotypes (13, 14): (i) cap SMCs, which are located in the subendothelial space and express contractile SMC proteins such as ACTA2; (ii) chondrocyte-like SMCs or chondromyocytes (17), which may promote calcification in the plaque and express chondrocyte markers (SOX9, COL2A1); (iii) fibroblast-like SMCs or fibromyocytes (18), which provide tensile strength and plaque stability by producing collagens and fibronectin and express fibroblast markers (LUM, BGN, DCN, OPG); and (iv) lipid-loaded SMCs or inflammatory SMCs (14), which accumulate lipids and take part in the inflammatory state of the plaque by producing pro-inflammatory cytokines. Because these last SMC-derived cells show expression of myeloid markers such as CD68, some authors have speculated about smooth muscle-to-macrophage differentiation in the plaque (19). However, there is a growing body of evidence challenging this theory (18, 20).

Misra et al. found that a few medial SMCs migrate into the intima at the shoulder of the growing plaque, proliferate, and first give rise to fibrous cap SMCs that express ACTA2 and PDGFRB (platelet-derived growth factor receptor beta). Subsequently, cap SMCs produce progeny that migrate into the core, downregulate SMC lineage markers, and differentiate into alternative mesenchymal phenotypes that promote lesion growth (15). Alencar et al. demonstrated that around two-thirds of SMC-derived cells express or have passed through a modulated state expressing LGALS3 (galectin 3) during their investment into the lesion. However, most cap SMCs do not pass through this intermediate stage (21). Collectively, these findings suggest that cap SMCs originate from medial SMCs that migrate to the subendothelial space without passing through the highly modulated phenotypes characteristic of SMC-derived cells in the plaque interior.

The separation in pathways leading to cap SMCs and modulated SMC-derived cells has significant implications for anti-atherosclerotic therapies. Targeting the entire SMC population in atherosclerosis may compromise SMC investment into the fibrous cap, leading to reduced cap thickness, and increased risk of thrombotic complications (15, 22, 23). However, strategies that target modulated SMC progeny in the lesion core could potentially represent a viable therapeutic approach, reducing plaque size without compromising fibrous cap integrity (21). Therefore, identifying the mechanisms that determine different SMC fates in plaque and how interventions might affect the balance between plaque-stabilizing and plaque-destabilizing SMC states will be a key area for exploration in the future.

It should be noted that the mechanisms of cap formation in humans may differ from those of mice. Human lesions preferentially form in regions of the arterial tree where the arterial intima has a large pre-existing population of SMCs (24). These intimal SMCs constitute the initial cap tissue and are the most likely sources of the fibrous cap throughout lesion development (6). Furthermore, it remains unknown whether human cap SMCs, like murine, are the product of oligoclonal proliferation. Studies of X chromosome inactivation patterns in homogenates of micro-dissected plaques from women with heterozygous polymorphisms on X-encoded genes provided evidence that the SMC population in the cap contains large clones (25, 26). However, subsequent studies found that such clones also exist in normal arteries raising the question of whether the clonal expansion occurs during artery development, physiological intimal thickening, or atherosclerosis (26, 27). New approaches are needed to settle this question in upcoming years.

### 2.2. Other cellular sources for cap SMCs

Cap SMCs in mice are primarily derived from local medial SMCs that undergo clonal expansion (13, 14, 28–30). However, when recruitment of medial SMCs is impaired, additional sources of cap SMCs may become quantitatively important, such as endothelial-to-mesenchymal transition of surface endothelial cells (31). One may speculate that the existence of these alternative mechanisms would increase the resilience of the cap SMC layer to thinning, but the reality is that thin-cap fibroatheromas still form and cause clinical events (7). This raises several questions: Do this alternative source of cap SMCs also contribute quantitatively to human fibrous caps? What are the cues necessary to initiate the transitioning process? Can it be forced to strengthen the fibrous cap and ultimately prevent plaque rupture? These are important questions for future research.

### 2.3. Recapitulation of embryonic pathways in SMCs during atherosclerosis

In normal arteries, the endothelium is positioned adjacent to a supporting layer of medial SMCs, which provides mechanical stability to the wall and enables the endothelium to regulate arterial diameter for optimal blood flow. The accumulation of plaque material in the intima disrupts this symbiotic relationship. Being a layered SMC structure in the subendothelial space, it is reasonable to consider that the cap is essentially a neo-media that substitutes for the lost connection to the arterial media. This interpretation is supported by the finding that three embryonic artery developmental programs are reactivated to recruit SMCs to form the fibrous cap: NOTCH, PDGF (platelet-derived growth factor), and TCF21 (transcription factor 21) signaling (Figure 1B).

Embryonic medial SMC differentiation relies on Notch signaling, which guides local mesenchymal precursors toward adopting SMC fate while suppressing the osteochondrogenic program (32, 33). In the development of the aortic arch, the expression of JAG1 (jagged canonical Notch ligand 1) on the endothelium triggers Notch signaling in neighboring neural crest-derived mesenchymal cells, initiating the formation of a subendothelial layer of SMCs in the maturing artery. The recruited SMCs themselves express JAG1 and can thereby propagate sequential waves of SMC differentiation leading to a multilayered arterial media (33). Notably, JAG1 and NOTCH3 (the typical Notch receptor in SMCs) are also expressed in cap cells of human and murine fibroatheromas, suggesting that Notch signaling could orchestrate cap formation through a similar mechanism (30). This was addressed in murine atherosclerosis by blocking Notch signaling in SMCs, which effectively reduced their ability to produce ACTA2 + cap SMCs (30). Notably, loss of Notch signaling did not affect the total recruitment or SMC-derived cells in plaques or the modulation to other SMC-derived subtypes, and gain-of-function experiments indicated that reduced Notch signaling is even required for proliferation and phenotypic modulation in murine atherosclerosis (30). Precise regulation of the Notch signaling cascade thus appears necessary to build the cap SMC population. This involves sequential loss of Notch signaling (for SMCs to exit the media, proliferate, and accumulate in the forming plaque) followed by a gain of Notch signaling (to acquire the SMC identity in the cap), in a process that bears similarities to arterial medial development.

PDGF signaling is also key for the radial construction of the developing arterial wall in murine and avian embryos (34–36). PDGFB (platelet-derived growth factor subunit B), which is secreted by nascent endothelial tubes, recruits and stimulates PDGFRB + mural cell precursors to mature into successive layers of SMCs from the inside out. As the SMCs mature, the expression of PDGFRB is downregulated, and they acquire lineage SMC markers such as ACTA2, TAGLN (transgelin), and MYH11 (34). Notably, PDGFRB is re-expressed in early cap SMCs in murine atherosclerotic plaques (15, 37). PDGF acts as a chemoattractant and mitogen for SMCs (38, 39), and persistent PDGF signaling through PDGFRB is necessary for SMC investment and retention within the fibrous cap (31, 40). Mechanistically, PDGF promotes SMC migration and transitioning to an extracellular matrix-synthetic state through metabolic reprogramming, thus playing a dual role in cap formation (31, 39). Blocking PDGF signaling leads to alternative sources of cap SMCs through an endothelial-to-mesenchymal-transition mechanism driven by IL1B (interleukin 1 beta) and TGFB (transforming growth factor beta). However, these alternative sources cannot sustain fibrous cap formation and maintenance within advanced atherosclerotic lesions (31), highlighting the relevance of the PDGF pathway for stable cap formation.

TCF21 is a transcription factor that controls cell fate decisions in the developing epicardium. TCF21 shifts epicardial progenitor cells away from the SMC lineage and is switched off in coronary vascular SMCs, but persistently expressed in epicardium-derived cardiac fibroblasts (41, 42). However, upon vascular injury, TCF21 is re-expressed in vascular wall SMC precursors and promotes SMC proliferation, migration, and phenotypic modulation towards a fibroblast gene expression program (18). TCF21 + cells are found in the fibrous cap of advanced human and murine atherosclerotic lesions (43). TCF21 inhibits SMC differentiation by blocking the myocardin-serum response factor pathway (44). Deletion of TCF21 in the SMC lineage reduces its ability to undergo phenotypic switching and decreases the proportion of SMC lineage cells in the fibrous cap (18). Altogether, TCF21 expression in the adult vascular wall might be a trait of a SMC precursor cell that, in a pathological setting, shifts SMCs away from their mature, contractile function towards a more fibroblast-like phenotype and contributes to forming the fibrous cap.

## 3. Impact of therapies on the fibrous cap

Therapies that strengthen fibrous caps have been a clear goal for researchers since plaque rupture was discovered as the main underlying cause of myocardial infarction. Table 1 shows a non-exhaustive list of current therapies and interventions in human or murine experimental models that have been reported to affect the fibrous cap thickness or SMC content. The targets are varied and include plasma cholesterol and glucose, SMCs, inflammation, the extracellular matrix, and cellular senescence.

**Table 1:** Impact of current therapies and interventions on fibrous cap state.

Intervention	Specie	Target	General effect	Effect in fibrous cap	Evaluation by	Reference
Statin treatment in patients with acute coronary syndrome	Human	3-hydroxy-3-methylglutaryl coenzyme A (HMG-CoA) reductase	Decreased cholesterol synthesis and ultimately reduced LDL levels in blood	Increased fibrous cap thickness in coronary and carotid plaques	Optical coherence tomography	(45)
Treatment of acute coronary syndrome patients with evolocumab	Human	Proprotein convertase subtilisin/kexin type 9 (PCSK9)	Increased LDL receptors on hepatic cells and thus decreased LDL circulating levels	Increased fibrous cap thickness and reduced macrophage infiltration in coronary and carotid plaques	Optical coherence tomography	(46)
Treatment of type 2 diabetes patients with ischemic heart disease with SGLT2 inhibitors	Human	Sodium-glucose cotransporter 2 (SGLT2)	Reduced glycemia and inflammatory state	Increased fibrous cap thickness	Optical coherence tomography	(47)
				Reduction in lipid and macrophage content		
Inactivation of Notch signaling in SMCs in PCSK9-expressing mice after 20 weeks of high fat diet (HFD)	Mouse	Notch signaling in smooth muscle cells	Reduced SMC investment in lesions	Reduced number of ACTA2 + cells in the fibrous cap	Histological and immunohistochemical analysis	(30)
(a) Knockout of *Pdgfrb* in SMCs or (b) pharmacological inhibition (imatinib) of PDGFRB signaling in *ApoE*-/- mice after 26 or 18 weeks of HFD, respectively	Mouse	PDGFRB signaling in smooth muscle cells	Inhibition of SMC recruitment and proliferation	(a) Reduced collagen and SMC lineage cells in the fibrous cap and increased intraplaque hemorrhage	Histological and immunohistochemical analysis	(31)
				(b) Reduced SMC lineage cells in the fibrous cap		
Knockout of *Tcf21* in SMCs in *ApoE*-/- mice after 16 weeks of HFD	Mouse	TCF21 signaling in smooth muscle cells	Blockade of TCF21-dependent modulation into a fibroblast-like phenotype	Reduced numbers of SMC lineage cells in the fibrous cap	Histological and immunohistochemical analysis	(18)
Knockout of *Klf4* in SMCs in *ApoE*-/- mice after 18 weeks of HFD	Mouse	KLF4 signaling ni smooth muscle cells	Disruption of KLF4 -dependent phenotypic modulation	Smaller lesions, thicker fibrous caps, increased plaque stability indices, and higher numbers of ACTA2 + cells in the fibrous cap	Histological and immunohistochemical analysis	(12)
Knockout of *Oct4* in SMCs in *ApoE*-/- mice after 18 weeks of HFD	Mouse	OCT4 signaling in smooth muscle cells	Reduced SMC investment in lesions	Bigger lesions, decreased plaque stability indices, and fibrous caps with reduced collagen and fewer ACTA2 + cells	Histological and immunohistochemical analysis	(22)
(a) Knockout of *Il1r1* in SMCs or (b) treatment with a neutralizing IL1B antibody in hyperlipidemic *ApoE*-/- mice	Mouse	IL1B signaling in smooth muscle cells	Reduced SMC investment and outward remodeling in lesions	Less SMCs, less collagen, and more anti-inflammatory macrophages (ARG1 + cells) in the fibrous cap	Histological and immunohistochemical analysis	(48)
Treatment of hyperlipidemic *ApoE*-/- mice with a neutralizing CXCL10 antibody	Mouse	CXCL10 signaling	Reduces the activation and recruitment of inflammatory cells	Increased collagen and ACTA2 + cells in the plaque	Histological and immunohistochemical analysis	(49)
Overexpression of MMP9 in macrophages of *ApoE*-/- mice	Mouse	Macrophage proteolytic activity	Increased extracellular matrix degradation	Increased features of plaque rupture	Histological and immunohistochemical analysis	(50)
Treatment with navitoclax in hyperlipidemic *Ldlr*-/- mice	Mouse	Anti-apoptotic BCL-2 pathways	Clearance of senescent cells	Enhanced fibrous cap thickness and more elastin and ACTA2 + cells in the fibrous cap	Histological and immunohistochemical analysis	(51)

Studies in humans have used intravascular optical coherence tomography to measure cap thickness and have found that both cholesterol-lowering (45, 46) and SGLT2 (sodium-glucose cotransporter 2) inhibition (47) may increase cap thickness (distance from lumen to a necrotic core). Whether this results from strengthening cap formation or less degradation is not known. Anti-inflammatory therapies, such as IL1B antagonism and colchicine, reduce the incidence of clinical events in patients with advanced atherosclerosis (52, 53), but whether this is achieved partly by increasing cap thickness is also unknown. Curiously, inhibition of IL1B in murine atherosclerosis inhibited cap formation (48). If this effect is conserved in human atherosclerosis, it could partly offset the beneficial effect of dampening macrophage-driven inflammation. Another potential modifier of fibrous cap thickness in patients is imatinib, which is a tyrosine kinase inhibitor used for the treatment of chronic myeloid leukemia that inhibits PDGFRB and limits cap formation in mice (31).

## 4. Discussion

Cells deriving from SMCs have complex roles in atherosclerosis driving plaque growth through modulation and proliferation and stabilizing the formed plaque by forming the fibrous cap. More research into the mechanisms that control the fate decisions of SMC-derived cells is needed to inform the development of therapies that can reduce plaque growth without jeopardizing the mechanical resilience of the plaque to rupture.

Recent findings in animal models suggest that the fibrous cap forms by a process resembling arterial media development. First, the cap has the structure of a media, consisting of layers of SMCs with contractile protein expression that provide mechanical support for the plaque endothelium. Therefore, the fibrous cap of atherosclerotic lesions may be thought of as a neo-media, restoring the junction between the SMCs and the endothelium that is temporarily lost in early atherosclerosis due to the accumulation of lipids and inflammatory cells in the arterial intima. It should be noted, however, that although medial SMCs do not transition through the intermediate LGAS3 + state to give rise to cap cells (21), some degree of phenotypic modulation is required (i.e., transient downregulation of NOTCH signaling) (30). Nevertheless, the phenotypic changes in cap SMCs are much more subtle than the ones experienced by the highly modulated SMC-derived phenotypes found in the lesion core, which, contrary to cap SMCs, do transition through an intermediate LGALS3 + state (21). This is in line with the observation that the cap SMCs do not show the full medial SMC phenotype, having lower levels of contractile protein and higher levels of extracellular matrix production. Furthermore, it is unknown whether the cap can contract and whether the normal endothelial-to-SMC crosstalk is reestablished. Second, fibrous cap formation requires reactivation of embryonic pathways seen in arterial media development, which are otherwise dormant in adult vascular SMCs. It is tempting to speculate that other pathways that orchestrate vascular SMC formation and differentiation during development (i.e., BMP9-10/SMAD7, WNT/b-Catenin) could also have an impact in fibrous cap formation (54, 55).

In the coming years, research on cap SMCs is anticipated to generate significant progress. By uncovering the molecular and cellular processes that govern SMC fate, migration and extracellular matrix production, scientists may identify novel therapeutic targets for preventing cap thinning and plaque rupture. Furthermore, advancements in imaging techniques, molecular profiling, gene editing technologies, and regenerative medicine may offer innovative approaches to modulate SMC behavior and promote cap stabilization, thereby providing new avenues for more effective atherosclerosis treatment.

## References

[1] Vaduganathan M Mensah GA Turco JV Fuster V Roth GA. The global burden of cardiovascular diseases and risk: a compass for future health. J Am Coll Cardiol. (2022) 80:2361–71. 10.1016/j.jacc.2022.11.005 36368511

[2] Falk E Nakano M Bentzon JF Finn AV Virmani R. Update on acute coronary syndromes: the pathologists’ view. Eur Heart J. (2013) 34:719–28. 10.1093/eurheartj/ehs411 23242196

[3] Bentzon JF Otsuka F Virmani R Falk E. Mechanisms of plaque formation and rupture. Circ Res. (2014) 114:1852–66. 10.1161/CIRCRESAHA.114.302721 24902970

[4] Stary HC. Atlas of atherosclerosis: progression and regression. 2nd ed. London: Parthenon Pub. Group (2003). p. 108.

[5] Schaar J. Terminology for high-risk and vulnerable coronary artery plaques. Eur Heart J. (2004) 25:1077–82. 10.1016/j.ehj.2004.01.002 15191780

[6] Stary HC Chandler AB Dinsmore RE Fuster V Glagov S Insull W A definition of advanced types of atherosclerotic lesions and a histological classification of atherosclerosis: a report from the Committee on Vascular Lesions of the Council on Arteriosclerosis, American Heart Association. Circulation. (1995) 92:1355–74. 10.1161/01.CIR.92.5.1355 7648691

[7] Burke AP Farb A Malcom GT Liang Y Smialek J Virmani R. Coronary risk factors and plaque morphology in men with coronary disease who died suddenly. N Engl J Med. (1997) 336:1276–82. 10.1056/NEJM199705013361802 9113930

[8] Grootaert MOJ Moulis M Roth L Martinet W Vindis C Bennett MR Vascular smooth muscle cell death, autophagy and senescence in atherosclerosis. Cardiovasc Res. (2018) 114:622–34. 10.1093/cvr/cvy007 29360955

[9] Li T Li X Feng Y Dong G Wang Y Yang J. The role of matrix metalloproteinase-9 in atherosclerotic plaque instability. Mediators Inflamm. (2020) 2020:3872367. 10.1155/2020/3872367 33082709 PMC7557896

[10] Newby AC. Metalloproteinase expression in monocytes and macrophages and its relationship to atherosclerotic plaque instability. Arterioscler Thromb Vasc Biol. (2008) 28:2108–14. 10.1161/ATVBAHA.108.173898 18772495

[11] Yurdagul A. Crosstalk between macrophages and vascular smooth muscle cells in atherosclerotic plaque stability. ATVB. (2022) 42:372–80. 10.1161/ATVBAHA.121.316233 PMC8957544 35172605

[12] Shankman LS Gomez D Cherepanova OA Salmon M Alencar GF Haskins RM KLF4-dependent phenotypic modulation of smooth muscle cells has a key role in atherosclerotic plaque pathogenesis. Nat Med. (2015) 21:628–37. 10.1038/nm.3866 25985364 PMC4552085

[13] Chappell J Harman JL Narasimhan VM Yu H Foote K Simons BD Extensive proliferation of a subset of differentiated, yet plastic, medial vascular smooth muscle cells contributes to neointimal formation in mouse injury and atherosclerosis models. Circ Res. (2016) 119:1313–23. 10.1161/CIRCRESAHA.116.309799 27682618 PMC5149073

[14] Jacobsen K Lund MB Shim J Gunnersen S Füchtbauer E-M Kjolby M Diverse cellular architecture of atherosclerotic plaque derives from clonal expansion of a few medial SMCs. JCI Insight. (2017) 2:e95890. 10.1172/jci.insight.95890 28978793 PMC5841865

[15] Misra A Feng Z Chandran RR Kabir I Rotllan N Aryal B Integrin beta3 regulates clonality and fate of smooth muscle-derived atherosclerotic plaque cells. Nat Commun. (2018) 9:2073. 10.1038/s41467-018-04447-7 29802249 PMC5970166

[16] Worssam MD Lambert J Oc S Taylor JCK Taylor AL Dobnikar L Cellular mechanisms of oligoclonal vascular smooth muscle cell expansion in cardiovascular disease. Cardiovasc Res. (2022) 138:1279–94. 10.1093/cvr/cvac138 PMC10202649 35994249

[17] Cheng P Wirka RC Kim JB Kim H-J Nguyen T Kundu R Smad3 regulates smooth muscle cell fate and mediates adverse remodeling and calcification of the atherosclerotic plaque. Nat Cardiovasc Res. (2022) 1:322–33. 10.1038/s44161-022-00042-8 36246779 PMC9560061

[18] Wirka RC Wagh D Paik DT Pjanic M Nguyen T Miller CL Atheroprotective roles of smooth muscle cell phenotypic modulation and the TCF21 disease gene as revealed by single-cell analysis. Nat Med. (2019) 25:1280–9. 10.1038/s41591-019-0512-5 31359001 PMC7274198

[19] Feil S Fehrenbacher B Lukowski R Essmann F Schulze-Osthoff K Schaller M Transdifferentiation of vascular smooth muscle cells to macrophage-like cells during atherogenesis. Circ Res. (2014) 115:662–7. 10.1161/CIRCRESAHA.115.304634 25070003

[20] Swirski FK Nahrendorf M. Do vascular smooth muscle cells differentiate to macrophages in atherosclerotic lesions? Circ Res. (2014) 115:605–6. 10.1161/CIRCRESAHA.114.304925 25214571 PMC4166538

[21] Alencar GF Owsiany KM Karnewar S Sukhavasi K Mocci G Nguyen AT Stem cell pluripotency genes Klf4 and Oct4 regulate complex SMC phenotypic changes critical in late-stage atherosclerotic lesion pathogenesis. Circulation. (2020) 142:2045–59. 10.1161/CIRCULATIONAHA.120.046672 32674599 PMC7682794

[22] Cherepanova OA Gomez D Shankman LS Swiatlowska P Williams J Sarmento OF Activation of the pluripotency factor OCT4 in smooth muscle cells is atheroprotective. Nat Med. (2016) 22:657–65. 10.1038/nm.4109 27183216 PMC4899256

[23] Kabir I Zhang X Dave JM Chakraborty R Qu R Chandran RR The age of bone marrow dictates the clonality of smooth muscle-derived cells in atherosclerotic plaques. Nat Aging. (2023) 3:64–81. 10.1038/s43587-022-00342-5 36743663 PMC9894379

[24] Stary HC Blankenhorn DH Chandler AB Glagov S Insull W Richardson M A definition of the intima of human arteries and of its atherosclerosis-prone regions. A report from the committee on vascular lesions of the council on arteriosclerosis, American heart association. Circulation. (1992) 85:391–405. 10.1161/01.cir.85.1.391 1728483

[25] Benditt EP. Evidence for a monoclonal origin of human atherosclerotic plaques and some implications. Circulation. (1974) 50:650–2. 10.1161/01.cir.50.4.650 4419679

[26] Murry CE Gipaya CT Bartosek T Benditt EP Schwartz SM. Monoclonality of smooth muscle cells in human atherosclerosis. Am J Pathol. (1997) 151:697–705. 9284818 PMC1857839

[27] Chung I-M Schwartz SM Murry CE. Clonal architecture of normal and atherosclerotic aorta. Am J Pathol. (1998) 152:913–23. 9546352 PMC1858235

[28] Bentzon JF Sondergaard CS Kassem M Falk E. Smooth muscle cells healing atherosclerotic plaque disruptions are of local, not blood, origin in apolipoprotein E knockout mice. Circulation. (2007) 116:2053–61. 10.1161/CIRCULATIONAHA.107.722355 17938286

[29] Bentzon JF Weile C Sondergaard CS Hindkjaer J Kassem M Falk E. Smooth muscle cells in atherosclerosis originate from the local vessel wall and not circulating progenitor cells in ApoE knockout mice. ATVB. (2006) 26:2696–702. 10.1161/01.ATV.0000247243.48542.9d 17008593

[30] Martos-Rodríguez CJ Albarrán-Juárez J Morales-Cano D Caballero A MacGrogan D de la Pompa JL Fibrous caps in atherosclerosis form by notch-dependent mechanisms common to arterial media development. ATVB. (2021) 41:e427–2439. 10.1161/ATVBAHA.120.315627 34261328

[31] Newman AAC Serbulea V Baylis RA Shankman LS Bradley X Alencar GF Multiple cell types contribute to the atherosclerotic lesion fibrous cap by PDGFRβ and bioenergetic mechanisms. Nat Metab. (2021) 3:166–81. 10.1038/s42255-020-00338-8 33619382 PMC7905710

[32] Briot A Jaroszewicz A Warren CM Lu J Touma M Rudat C Repression of Sox9 by Jag1 is continuously required to suppress the default chondrogenic fate of vascular smooth muscle cells. Dev Cell. (2014) 31:707–21. 10.1016/j.devcel.2014.11.023 25535917 PMC4311887

[33] Manderfield LJ High FA Engleka KA Liu F Li L Rentschler S Notch activation of jagged1 contributes to the assembly of the arterial wall. Circulation. (2012) 125:314–23. 10.1161/CIRCULATIONAHA.111.047159 22147907 PMC3260393

[34] Greif DM Kumar M Lighthouse JK Hum J An A Ding L Radial construction of an arterial wall. Dev Cell. (2012) 23:482–93. 10.1016/j.devcel.2012.07.009 22975322 PMC3500096

[35] Mellgren AM Smith CL Olsen GS Eskiocak B Zhou B Kazi MN Platelet-derived growth factor receptor β signaling is required for efficient epicardial cell migration and development of two distinct coronary vascular smooth muscle cell populations. Circ Res. (2008) 103:1393–401. 10.1161/CIRCRESAHA.108.176768 18948621 PMC2757035

[36] Tomanek RJ Hansen HK Christensen LP. Temporally expressed PDGF and FGF-2 regulate embryonic coronary artery formation and growth. Arterioscler Thromb Vasc Biol. (2008) 28:1237–43. 10.1161/ATVBAHA.108.166454 18420995 PMC2748328

[37] Raines EW. PDGF and cardiovascular disease. Cytokine Growth Factor Rev. (2004) 15:237–54. 10.1016/j.cytogfr.2004.03.004 15207815

[38] Davies MG Owens EL Mason DP Lea H Tran PK Vergel S Effect of platelet-derived growth factor receptor-α and -β blockade on flow-induced neointimal formation in endothelialized baboon vascular grafts. Circ Res. (2000) 86:779–86. 10.1161/01.RES.86.7.779 10764412

[39] Gerthoffer WT. Mechanisms of vascular smooth muscle cell migration. Circ Res. (2007) 100:607–21. 10.1161/01.RES.0000258492.96097.47 17363707

[40] Sano H Sudo T Yokode M Murayama T Kataoka H Takakura N Functional blockade of platelet-derived growth factor receptor-β but not of receptor-α prevents vascular smooth muscle cell accumulation in fibrous cap lesions in apolipoprotein E-deficient mice. Circulation. (2001) 103:2955–60. 10.1161/01.CIR.103.24.2955 11413086

[41] Acharya A Baek ST Huang G Eskiocak B Goetsch S Sung CY The bHLH transcription factor Tcf21 is required for lineage-specific EMT of cardiac fibroblast progenitors. Development. (2012) 139:2139–49. 10.1242/dev.079970 22573622 PMC3357908

[42] Braitsch CM Combs MD Quaggin SE Yutzey KE. Pod1/Tcf21 is regulated by retinoic acid signaling and inhibits differentiation of epicardium-derived cells into smooth muscle in the developing heart. Dev Biol. (2012) 368:345–57. 10.1016/j.ydbio.2012.06.002 22687751 PMC3414197

[43] Nurnberg ST Cheng K Raiesdana A Kundu R Miller CL Kim JB Coronary artery disease associated transcription factor TCF21 regulates smooth muscle precursor cells that contribute to the fibrous cap. PLoS Genet. (2015) 11:e1005155. 10.1371/journal.pgen.1005155 26020946 PMC4447275

[44] Nagao M Lyu Q Zhao Q Wirka RC Bagga J Nguyen T Coronary disease-associated gene TCF21 inhibits smooth muscle cell differentiation by blocking the myocardin-serum response factor pathway. Circ Res. (2020) 126:517–29. 10.1161/CIRCRESAHA.119.315968 31815603 PMC7274203

[45] Ozaki Y Garcia-Garcia HM Beyene SS Hideo-Kajita A Kuku KO Kolm P Effect of statin therapy on fibrous cap thickness in coronary plaque on optical coherence tomography—review and meta-analysis. Circ J. (2019) 83:1480–8. 10.1253/circj.CJ-18-1376 31118354

[46] Yano H Horinaka S Ishimitsu T. Effect of evolocumab therapy on coronary fibrous cap thickness assessed by optical coherence tomography in patients with acute coronary syndrome. J Cardiol. (2020) 75:289–95. 10.1016/j.jjcc.2019.08.002 31495548

[47] Sardu C Trotta MC Sasso FC Sacra C Carpinella G Mauro C SGLT2-inhibitors effects on the coronary fibrous cap thickness and MACEs in diabetic patients with inducible myocardial ischemia and multi vessels non-obstructive coronary artery stenosis. Cardiovasc Diabetol. (2023) 22:1–15. 10.1186/s12933-023-01814-7 37005586 PMC10067292

[48] Gomez D Baylis RA Durgin BG Newman AAC Alencar GF Mahan S Interleukin-1β has atheroprotective effects in advanced atherosclerotic lesions of mice. Nat Med. (2018) 24:1418–29. 10.1038/s41591-018-0124-5 30038218 PMC6130822

[49] Segers D Lipton JA Leenen PJM Cheng C Tempel D Pasterkamp G Atherosclerotic plaque stability is affected by the chemokine CXCL10 in both mice and humans. Int J Inflam. (2011) 2011:1–9. 10.4061/2011/936109 PMC3227498 22164344

[50] Gough PJ Gomez IG Wille PT Raines EW. Macrophage expression of active MMP-9 induces acute plaque disruption in apoE-deficient mice. J Clin Invest. (2006) 116:59–69. 10.1172/JCI25074 16374516 PMC1319218

[51] Childs BG Zhang C Shuja F Sturmlechner I Trewartha S Fierro Velasco R Senescent cells suppress innate smooth muscle cell repair functions in atherosclerosis. Nat Aging. (2021) 1:698–714. 10.1038/s43587-021-00089-5 34746803 PMC8570576

[52] Ridker PM Everett BM Thuren T MacFadyen JG Chang WH Ballantyne C Antiinflammatory therapy with canakinumab for atherosclerotic disease. N Engl J Med. (2017) 377:1119–31. 10.1056/NEJMoa1707914 28845751

[53] Tardif J-C Kouz S Waters DD Bertrand OF Diaz R Maggioni AP Efficacy and safety of low-dose colchicine after myocardial infarction. N Engl J Med. (2019) 381:2497–505. 10.1056/NEJMoa1912388 31733140

[54] Riascos-Bernal DF Chinnasamy P Cao LL Dunaway CM Valenta T Basler K β-Catenin C-terminal signals suppress p53 and are essential for artery formation. Nat Commun. (2016) 7:12389. 10.1038/ncomms12389 27499244 PMC4979065

[55] Wang L Rice M Swist S Kubin T Wu F Wang S BMP9 and BMP10 act directly on vascular smooth muscle cells for generation and maintenance of the contractile state. Circulation. (2021) 143(14):1394–410. 10.1161/CIRCULATIONAHA.120.047375 33334130

